# 

**Published:** 1900-02

**Authors:** 


### BOOKS RECEIVED.

A Manual of Surgical Treatment. By W. Watson Cheyne, M. B., F. R. C. S., F. R. S., Professor of Surgery in King's College, London, Surgeon to King's College Hospital, etc., and F. F. Burghard, M. D. and M S. (Lond.), F. R. C. S, Teacher of Practical Surgery in King's College, London, Surgeon to King's College Hospital, etc In six imperial 8vo volumes, with illustrations. Volume II, 382 pages with 141 illustrations. Cloth, $4.00 net. Philadelphia and New York : Lea Brothers & Co. 1899.

Lea's Series of Pocket Text-Books. Histology and Pathology. By John B. Nichols, M. D., Demonstrator of Histology, Medical Department Columbian University, and F. P. Vale, M. D., Assistant in Pathology, Medical Department University of Georgetown, Washington, D. C. In one handsome 12mo. volume of 452 pages, with 213 illustrations. Cloth, $1.75 net. Flexible red leather, $2.25 net. Philadelphia and New York : Lea Brothers & Co.

A Text-Book of Diseases of Women. By Charles B. Penrose, M. D., Ph. D., Professor of Gynecology in the University of Pennsylvania ; Surgeon to the Gynecean Hospital, Philadelphia. Octavo, pp. 531. Illustrated. Third edition, revised. Philadelphia : W. B. Saunders, 925 Walnut street. 1900.

Chirurgie du Foie et des Voies Biliaires, par J. Pantaloni (de Marseille), avec 348 figures dans le texte. Octavo, pp. 626. Paris : Institute de Bibliographie Scientifique, 93 Boulevard Saint-Germain. 1899.

The Treatment of Diseases of the Nervous System. A Manual for Practitioners. By Joseph Collins, M D. One volume, 8vo. Extra muslin, $5 00 net. William Wood & Company.

Dndley's Gynecology. A Treatise on the Principles and Practice of Gynecology. By E. C. Dudley, A. M., M. D , Professor of Gynecology in the Northwestern University Medical School, Chicago. New (2d) edition. In one very handsome 8vo volume of 717 pages, with 453 engravings, of which 47 are in colors and 8 colored plates. Just ready. Cloth, $5 00 net ; leather, $6.00 net.

The Principles and Practice of Modern Surgery. For the Use of Students and Practitioners of Medicine and Surgery By John B. Roberts, M. D., Professor of Anatomy and Surgery in the Philadelphia Polyclinic ; Mütter Lecturer on Surgical Pathology of the College of Physicians of Philadelphia. New (2d) and revised edition. In one very handsome 8vo volume of 838 pages, with 474 engravings and 8 plates in colors and monochrome. Cloth, $4 25 net ; leather, $5 25 net.

Christian Science : An Exposition of Mrs. Eddy's Wonderful Discovery, Including its Legal Aspects. A Plea for Children and Other Helpless Sick. By William A. Purrington, Lecturer in the University and Bellevue Hospital Medical College, and in the New York College of Dentistry, upon Law in Relation to Medical Peactice ; one of the authors of A System of Legal Medicine. New York : E B. Treat & Company. 1900.

A Manual of the Practice of Medicine, Prepared Especially for Students. By A. A. Stevens, A. M., M. D., Professor of Pathology in the Woman's Medical College of Pennsylvania ; Lecturer on Terminology and Instructor in Physical Diagnosis in the University of Pennsylvania ; Physician to St. Agnes's Hospital and Out-patient Department of the Episcopal Hospital, etc. Fifth edition, revised and enlarged. Illustrated. Philadelphia : W. B. Saunders. 1898

General and Local Anesthesia. By Aimé Paul Heineck, M. D , Clinical Instructor in Genito-Urinary Diseases College of Physicians and Surgeons, Chicago ; Clinical Instructor in Gynecology, Chicago Clinical School ; Clinical Instructor in Surgery, Northwestern University Woman's Medical College. 124 pages, $1.00. Chicago : G. P. Engelhard & Co., Publishers, 358-362 Dearborn street.

Recollections of a Rebel Surgeon and Other Sketches, or In the Doctor's Sappy Days. By F. E. Daniel, M. D. Illustrated. Austin, Texas : Von Boeckmann, Schultze & Co. 1899.

Children, Acid and Alkaline. "Health, the Golden Mean." The Law of Diet Selection, Contraria. The Therapeutic Law, Semilia. By Thomas C.

Duncan, M. D., Ph. D., LL. D., Professor of Medicine and Diseases of the Chest, Dunham Medical College; Consulting Physician to Chicago Foundling's Home, St. Anthony Hospital, Cook County Hospital, etc., etc. Philadelphia: Boericke & Tafel. 1900.

